# Hierarchical Au@Pt nanoparticle/amino benzoic acid polymer-based hybrid material for labeled and label-free detection of interleukin-6: a comparative assessment

**DOI:** 10.1007/s00604-024-06745-y

**Published:** 2024-10-21

**Authors:** Dayana Soto, Verónica Serafín, María Pedrero, José M. Pingarrón, Susana Campuzano, Jahir Orozco

**Affiliations:** 1 Max Planck Tandem Group in Nanobioengieneering, University of Antioquia, Complejo Ruta N, Calle 67 Nº 52-20, 050010 Medellín, Colombia; 2 Departamento de Química Analítica, Facultad de CC. Químicas, Universidad Complutense de Madrid, Pza. de Las Ciencias 2, 28040 Madrid, Spain; 3 Present Address: CECOLTEC Group, Cecoltec Services S.A.S Company, Universidad EAFIT, Edificio Ingenierías, Bloque 19, 050022 Medellín, Colombia

**Keywords:** Hybrid nanomaterial, Multi-walled carbon nanotubes, Electrochemical detection, Screen-printed carbon electrode, Differential pulse voltammetry, Gold nanoparticles, Label-free immunosensor, Sandwich-like immunosensor

## Abstract

Interleukin-6 (IL6) is a cytokine mainly involved in inflammatory processes associated with various diseases, from rheumatoid arthritis and pathogen-caused infections to cancer, where malignant cells exhibit high proliferation and overexpression of cytokines, including IL6. Furthermore, IL6 plays a fundamental role in detecting and differentiating tumor cells, including colorectal cancer (CRC) cells. Therefore, given its range of biological activities and pathological role, IL6 determination has been claimed for the diagnosis/prognosis of immune-mediated diseases. Herein, a comparative study is presented of labeled and label-free electrochemical immunosensors involving a hierarchical Au@Pt nanoparticle/polymer hybrid material for detecting IL6. The electrochemical immunosensors were independently coupled to the surface of screen-printed carbon electrodes (SPCEs) previously modified with polymeric layers. While in the label-free immunosensor, an anti-IL6 antibody (IL6-Ab) was covalently bound to the modified SPCE surface, in the sandwich-like amperometric immunosensor, an anti-biotinylated-IL6 antibody (B-IL6-Ab) was attached to the electrode through biotin-avidin affinity interactions. The label-free format employed a straightforward detection of IL6 by differential pulse voltammetry (DPV). The resulting electrochemical immunosensors exhibited a linear dynamic range from 50 to 750 pg/mL IL6, with detection limits (LOD) of 14.4 and 6.0 pg/mL for label-free and sandwich-like immunosensors, respectively. This outstanding performance makes them versatile platforms for clinical analysis of a panel of biomarkers for early diagnosis/prognosis of inflammatory processes associated with oncological diseases, among other pathologies.

**Supplementary Information:**

The online version contains supplementary material available at 10.1007/s00604-024-06745-y.

## Introduction

Cytokines are a family of proteins essential in regulating the immune response when developing various diseases, ranging from rheumatoid arthritis and autoimmune diseases to cancer and, more recently, COVID-19 [1, 2]. For example, IL6 is a glycosylated multifunctional cytokine that influences cancer cell activity and differentiation and immunomodulation of the microenvironment as an essential mediator of the acute inflammatory response [3–5]. IL6 glycoprotein is produced in various cells, including fibroblasts, endothelial cells, keratinocytes, macrophages, T cells, and mast cells. This glycoprotein is synthesized early in inflammation and delivered to the liver via the bloodstream, followed by rapid induction of a wide variety of acute-phase proteins, such as C-reactive protein (CRP), serum amyloid A (SAA), fibrinogen, and haptoglobin among others [3, 6]. In addition, previous reports showed that cancer patients have an elevated cytokine profile associated with an inflammatory response [3, 7]. Hence, this suggests that IL6 glycoprotein is released into body fluids and can be used as a biomarker in autoimmune and cancer disease diagnosis. For example, while median IL6 serum level in control cohorts was 1.31 pg/mL (range 0–37 pg/mL), the overall median of the reported cut-offs of serum IL6 was 10 pg/mL (range: 1.9–130 pg/mL) in cancer patients [8]. Moreover, according to the study of Ding et al., serum IL6 levels ranged from 4.27 to 123.71 pg/mL in systemic lupus erythematosus (SLE) patients and from 0.93 to 10.46 pg/mL in healthy controls [9].

The determination of IL6 has been performed using an enzyme-linked immunosorbent assay (ELISA) in human saliva, blood, and tissue samples [1, 10, 11]. Other alternative methodologies used chemiluminescent [2, 12], colorimetric assays [13, 14], surface-enhanced Raman spectroscopy (SERS) [15], electrochemical biosensors [16–18], and proteotronics exploring the electronic response of biomolecules by a complex network approach [19, 20]. However, ELISA has some limitations, including laborious procedures, limited multiplexing options, the need for centralized laboratory equipment, and the demand for large sample volumes, which limit the accuracy of target quantification and miniaturized outcomes [7, 21]. Likewise, the above-mentioned alternative methodologies are technically demanding and require sophisticated laboratory equipment and highly qualified personnel, restricting their practical feasibility in early diagnosis and screening. Therefore, sensitive, specific, simple, rapid, and accurate tests to detect IL6 in health and disease conditions are highly required for the timely diagnosis/prognosis of immune-mediated diseases.

The amplification of electrochemical signals is a powerful strategy to achieve clinically relevant LOD values [22, 23]. Therefore, distinct amplification methodologies have been reported for this purpose. For example, signal amplification that includes a combination of enzymatic amplification from enzymes or nanozymes [24–26] with the high catalytic activity and fast electron transfer provided by metallic nanoparticles (NPs) [27, 28] supported on nanostructured polymeric or carbonaceous surfaces. Among many possibilities, carbon nanotubes (CNTs) stand out as supporting materials due to their large surface area, high conductivity, and surface functionality for anchoring various biomolecules and nanomaterials [29]. Furthermore, these supporting platforms can be modified with highly conductive nanomaterials such as metal nanoparticles or quantum dots (QDs), among others, that, in synergy with enzymes, enhance the amplification capabilities of labels and, therefore, improve the analytical performance of the resulting electrochemical biosensors [30].

Herein, we report a comparative evaluation of the design-based synergy of properties of hierarchical hybrid nanomaterials from Au@Pt nanoparticles/polymers for label-free and sandwich-shaped determination of IL6. The label-free electrochemical immunosensor involves an anti-human IL6 antibody covalently immobilized onto an SPCE, previously functionalized with a thin *p*-aminobenzoic acid (pABA) polymeric layer incorporating Au@Pt nanoparticles. While the polymeric layers provided available carboxylic acid moieties for the antibody attachment, the hierarchical Au@Pt nanoparticles favored electron transfer between the redox probe and the electrode surface, thus enhancing the determination of IL6 by DPV. The sandwich-like electrochemical immunosensor involves neutravidin incorporated onto a pABA polymeric layer-modified electrode surface, which binds to a biotinylated antibody through affinity interactions to recognize the IL6 glycoprotein. Detection was done by chronoamperometry using an amplification label formed by a hybrid Au@Pt, detection antibody, and horseradish peroxidase (HRP) supported at multi-walled CNTs (MWCNTs). The resultant immunosensors were highly specific for IL6 and appropriate for its quantification in spiked commercial human serum samples, holding considerable potential for determining biomarkers associated with inflammatory processes. Also, the versatility and the low LOD value achieved with both immunoplatforms make them suitable for supporting disease diagnosis near the patient.

## Experimental

Details about apparatus and electrodes, reagents and solutions, nanobiohybrid synthesis and assembly of the immunosensors, optimization of experimental parameters, electrochemical measurements, evaluation of the analytical parameters, selectivity studies, quantification of IL6 in serum samples are provided in the Supporting Information.

## Results and discussion

### Development of the electrochemical immunosensors

**Scheme 1:**
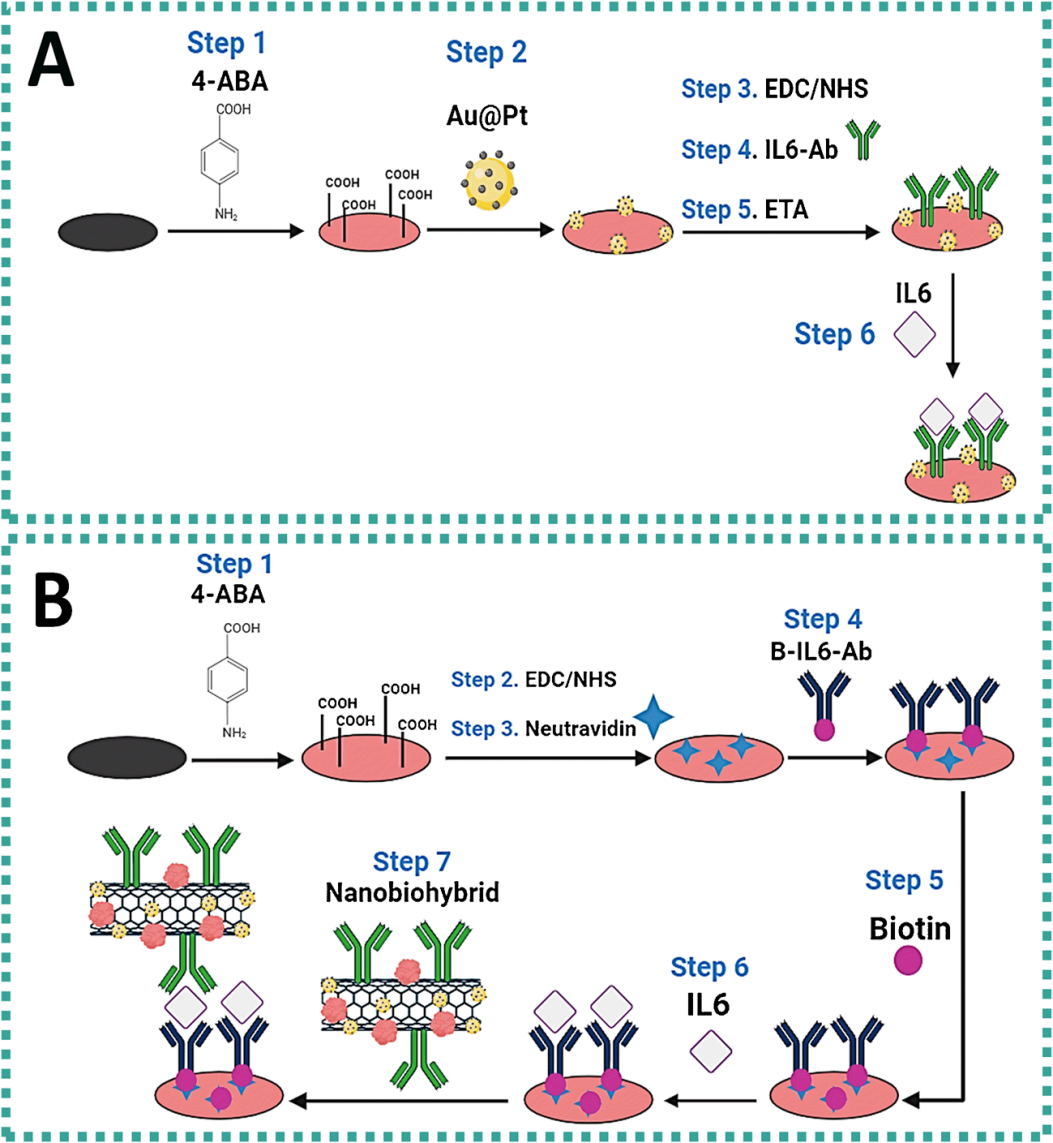
Assembly steps for the development of the electrochemical immunosensors. **A** Label-free immunosensor: (1) Polymeric layer formation. (2) Au@Pt electrodeposition. (3) Carboxylic acid activation. (4) Covalent antibody immobilization. (5) Blocking of remanent NHS-esters. (6) IL6 glycoprotein capture. **B** Sandwich-like immunosensor: (1) Polymeric layer formation. (2) Carboxylic acid activation. (3) Anchorage of neutravidin. (4) Biotinylated-antibody affinity immobilization. (5) Blocking of remanent NHS-esters and unspecific sites. (6) IL6 glycoprotein capture. (7) Hybrid label recognition

Label-free and sandwich-like electrochemical immunosensors for IL6 were developed, as shown in Scheme 1. Both immunosensors were assembled, step by step, forming a thin pABA layer onto the SPCE surface.

To prepare the label-free immunosensor (Scheme 1A), Au@Pt nanoparticles were electroplated on the previously formed thin polymeric layer with high electrochemical activity. The modification of SPCE surface was followed by the chemical activation of carboxylic acids with *N*-(3-dimethylaminopropyl)-*N*′-ethylcarbodiimide hydrochloride/*N*-hydroxysuccinimide (EDC/NHS), forming a reactive *N*-hydroxysuccinimidyl ester (NHS-ester) on the electrode surface suitable for covalent anchoring of biomolecules. After activation of carboxylic acids, IL6-Ab was attached by amide bond formation between the NHS-ester onto the SPCE surface and primary amine groups of the antibody. Thereafter, unreacted NHS-esters were blocked with ethanolamine (ETA), preventing the non-specific binding of proteins onto the SPCE surface. As a result, the IL6-Ab attached to the surface of SPCE recognized the target protein.

For the preparation of the sandwich-like electrochemical immunosensor, so-called because it uses two antibodies to detect the target analyte (Scheme 1B), the formation of the polymeric layer was followed by the chemical activation of carboxylic acids with a mixture of EDC/NHS, the covalent immobilization of neutravidin through activated NHS-ester, and the attachment of B-IL6-Ab by affinity interaction with the Fc region of the biotinylated antibody. After blocking free NHS-ester with biotin solutions, the B-IL6-Ab anchored to the surface of SPCE recognized the IL6 protein. Finally, the hierarchical nanobiohybrid was added for signal amplification.

### Morphology of MWCNTs/Au@Pt nanohybrid

**Fig. 1:**
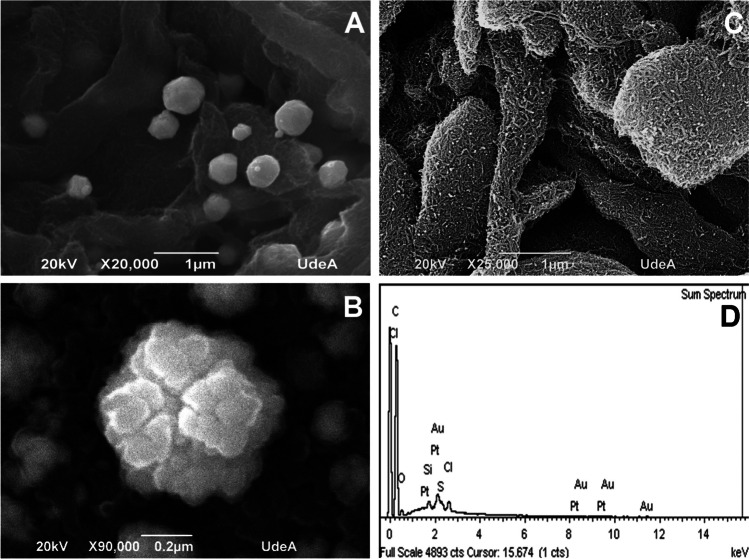
Characterization of the synthesized hybrid nanomaterial used as a label for the preparation of the IL6 immunosensor: SEM images of **A** MWCNTs/Au@Pt, **B** Au@Pt, **C** MWCNTs; **D** EDX analysis of MWCNTs/Au@Pt

Figure 1 a B, and C show SEM images of MWCNTs/Au@Pt, Au@Pt, and MWCNTs, respectively. The formation of a carbonaceous nanohybrid is evident in Fig. 1A. The images clearly show a particular hierarchical structure from Au@Pt nanoparticles embedded in the commercial MWCNTs whose tubular morphology has already been reported by other authors using TEM [31]. The nanoparticles are formed through the surfactant-assisted electrodeposition method. The difference in the reduction potentials of the two soluble metal salts (Au(III) and Pt(IV) species) and the presence of an F127 surfactant solution at low concentration play a key role in the one-step synthesis of the hierarchical structure of metallic nanoparticles. So, due to the different reduction potentials, the reduction of the Au ions preferentially occurs quickly to form the Au seeds on the surfactant template, followed by an overgrowth of dendritic Pt nanoparticles on the Au seeds, which SEM confirms. Interestingly, the nanoparticles’ size and the Pt layer’s thickness on the Au nuclei can be easily tuned by adjusting the precursor solutions’ Pt/Au molar ratio. Through optimization of precursor molar ratios and electrodeposition conditions, Au@Pt nanoparticles with enhanced activity can be formed, which is essential to improve the electron transfer in the resulting immunosensors [32, 33]. Figure 1 C corroborates the tubular-like morphology of the MWCNTs. As the parameters for forming the hybrid were optimized elsewhere [34], only the electrochemical response obtained by CV was optimized here. EDX elemental mapping measurements (Fig. 1D) showed the elemental distribution of Au/Pt nanoflowers and MWCNTs. Au and Pt were both homogeneously distributed on MWCNTs surface, confirming the formation of electrochemically synthesized Au@Pt nanoparticles with a high surface-to-volume ratio, thus providing numerous active sites for further modification and use [35].

### Electrochemical monitoring of the stepwise immunosensor preparation

**Fig. 2:**
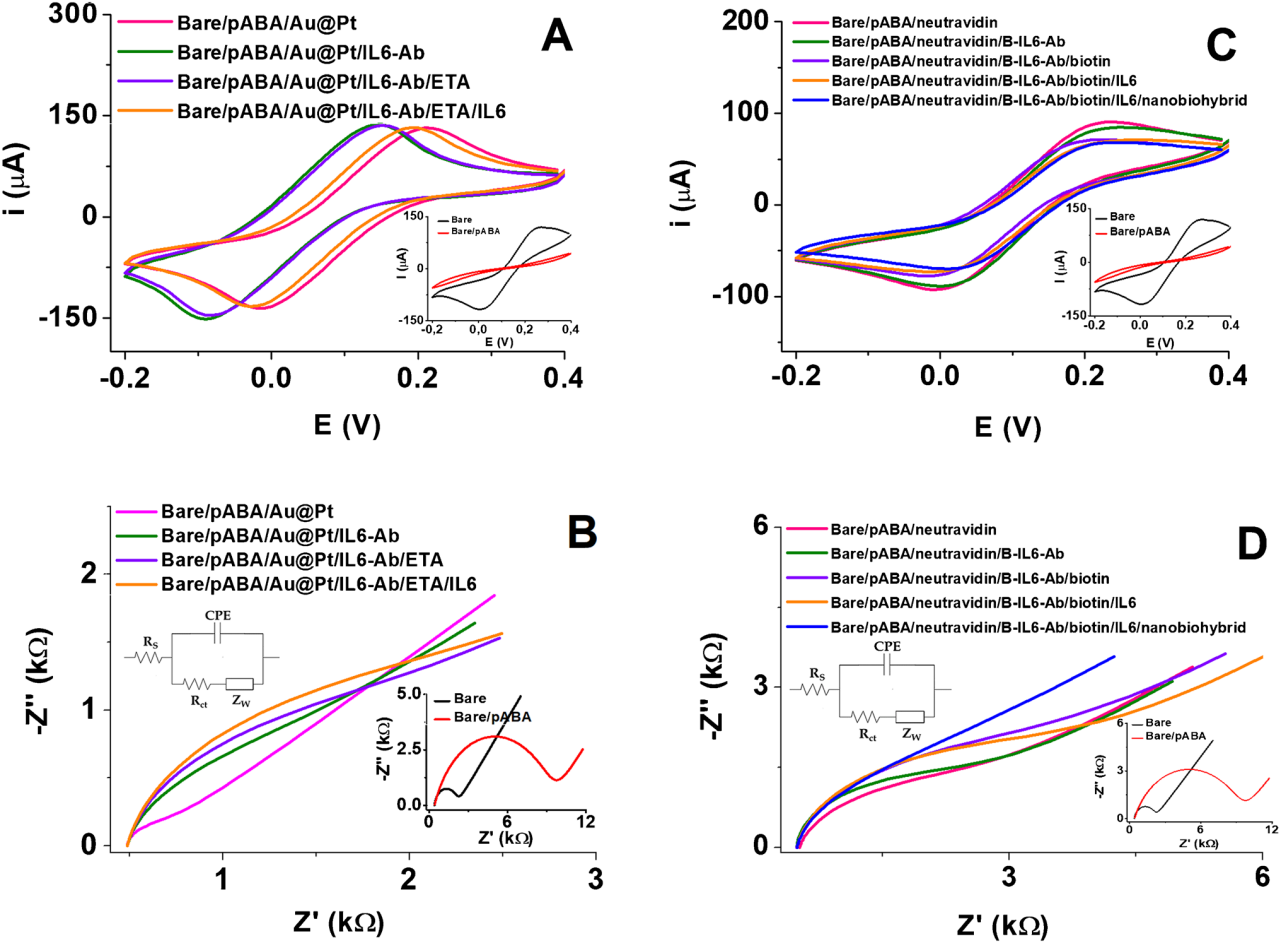
Electrochemical characterization of the label-free (**A**, **B**) and sandwich-like (**C**, **D**) immunosensing formats. **A**, **C** Cyclic voltammograms and **B**, **D** Nyquist plots recorded with 5 mM [Fe(CN)_6_].^4−/3−^/KNO_3_ redox probe at 100 kHz to 0.01 Hz frequency range and 10 mV amplitude. The bare and nanomaterial-modified electrode plots are in the insets of both figures. Inset of **B**, **D**: Randles equivalent circuit used to fit impedance measurements (Rs, resistance of electrolyte solution; *R*_ct_, charge transfer resistance; *Z*_*w*_, Warburg impedance; CPE, constant phase element)

Each step of the immunosensor assembly was electrochemically characterized by CV and EIS using 5 mM [Fe(CN)_6_]^4−/3−^/KNO_3_ as the redox probe (Fig. 2). The highest current intensity was observed for the bare SPCE because the redox probe diffused to the SPCE surface without steric hindrance and, therefore, with fast electron transfer kinetics. After the formation of the thin pABA polymeric layer on the SPCE surface, the current intensity decreased drastically due to electrostatic repulsion between the pABA’s carboxylic acids onto the SPCE surface and the redox probe in solution, as well as the limitation of the electron transfer caused by the steric hindrance of the polymeric layer (inset, Fig. 2 A and C). However, the incorporation of the Au@Pt nanoparticles provoked a noticeable current increase due to the catalytic properties of the nanoparticles (inset, Fig. 2A). Once carboxylic acids were activated and the IL6-Ab was attached to the SPCE surface, the current intensity dropped due to hindered electron transfer (inset, Fig. 2A). As expected, these interfacial changes were more evident when using EIS. This technique showed significant changes in charge transfer resistance after each immunosensor assembly step (Fig. 2B). The interfacial electrical changes evidenced the successful biomolecule immobilization onto the SPCE surface. The immobilized biomolecules hindered the electron transfer from the redox probe in the solution to the SPCE surface, thus increasing the *R*_ct_. The electrochemical process on the SPCE surface was modeled by the Randles equivalent circuit depicted in Fig. 1B (inset).

The specific molecular binding on the SPCE surface altered the interfacial electrical properties, and one or more electrical equivalent circuit elements changed significantly (e.g., *R*_ct_). Therefore, we used *R*_ct_ to characterize the anti-IL6-Ab-IL6 protein molecular binding on the SPCE surface. Table S1 in Supporting Information summarizes all fitted circuit elements. Figure 2 B and D inset show the Nyquist plot from the bare SPCE, which exhibited the typical trend of a conductive surface with a low charge transfer resistance (*R*_ct_ = 1707.9 ± 0.1 Ω) and a well-defined *Z*_*w*_ indicating the electrochemical reaction kinetics was controlled by probe diffusion. However, after the formation of the polymeric layer onto the SPCE surface, the Nyquist plot semicircle diameter increased significantly, indicating a more significant hindrance to electron transfer (*R*_ct_ = 8988.5 ± 0.2 Ω). This was attributed to the electrostatic repulsion between carboxylic acids on the SPCE surface and the negatively charged probe, as well as to the less conductive character of the polymeric layer. Once the Au@Pt nanoparticles were incorporated on the modified electrode (inset in Fig. 2B), the *R*_ct_ was significantly reduced to (104.5 ± 0.1) Ω; therefore, the electron transfer was promoted on the SPCE-modified surface.

In contrast, once carboxylic acids were activated and IL6-Ab covalently anchored on the SPCE surface in the label-free immunosensor (Fig. 2B), the *R*_ct_ increased to (1107.6 ± 0.1) Ω because this attached biomolecule hindered the electron transfer. This increase in *R*_ct_ indicated the successful attachment of the antibody on the SPCE surface. Blocking the remaining activated esters with ETA solution reduced the negative charges and introduced hydrophilic groups, slightly increasing *R*_ct_ to (1392.9 ± 0.2) Ω. When the antibody recognized the target protein, *R*_ct_ significantly increased from (1392.9 ± 0.2) Ω up to (1992.9 ± 0.1) Ω.

In the case of the sandwich-like immunosensor, the neutravidin bond, after activation of the carboxylic acid groups, increased current intensity probably attributed to the reorganization of the polymeric layer, favoring diffusion of the redox probe toward the electrode surface. However, the anchorage of B-IL6-Ab generated a slight decrease in current, most likely due to hindered electron transfer (Fig. 2C). After blocking unreacted NHS-esters, the current intensity decreased because the deactivation of unreacted NHS-esters increased electrostatic repulsion and limited electron transfer. Incorporating the nanobiohybrid increased the current intensity after the IL6 protein recognition due to the improved electrical properties of the nanobiohybrid with MWCNTs and Au@Pt (Fig. 2C).

Regarding the process monitorization by EIS, once carboxylic acids were activated and neutravidin anchored on the SPCE surface, the *R*_ct_ decreased to (1971.4 ± 0.1) Ω, consistent with that observed by CV because this biomolecule promoted reorganization of the polymeric layer decreasing negative charges and endorsing the transfer of electrons from the redox probe. Also, when the B-IL6-Ab was immobilized, the *R*_ct_ increased to (2012.7 ± 0.2) Ω. This slight increase in *R*_ct_ indicated the successful attachment of the antibody on the SPCE surface. Furthermore, the interaction of the remaining activated esters with biotin increased the coverage of the active electrode surface, increasing *R*_ct_ to (2945.2 ± 0.1) Ω. When the antibody recognized the target protein, *R*_ct_ significantly decreased from (3082.9 ± 0.1) Ω down to (2062.1 ± 0.3) Ω upon the nanobiohybrid interaction. This result indicates that the successful antibody-protein molecular biorecognition event hindered the electron transfer at the SPCE/electrolyte interface and the improvement of the electrical properties imparted by the nanobiohybrid. Furthermore, the values of the chi-squared function (*χ*^2^) were lower than 2.79 × 10^4^, indicating a proper fitting of experimental data and the electrical equivalent circuit (see Table S1 in the Supporting Information).

### Evaluation of critical experimental variables

Hybrid nanoparticles were prepared by electrosynthesis based on the method of seed-mediated growth [36, 37]. However, the conditions for the development of the label-free immunosensor were evaluated. These conditions included the concentration of IL6-Ab and the incubation time with activated SPCE/pABA for immobilization, type of activation mixture, blocking reagent and incubation time for forming the immunosensor, IL6 protein recognition, and preparation of the label-free immunosensor. Figs. S1 and S2 (in the Supporting Information) show DPV responses recorded upon increasing concentrations of IL6-Ab in the presence of 600 pg/mL (signal S) or absence (blank signal, B) of standard IL6, as well as the corresponding S/B ratio. Higher S/B ratios were selected as the criterion for choosing the value of each variable for further work. Fig. S1A shows a higher S/B ratio for 100 μg/mL of IL6-Ab solution incubated for 60 min (Fig. S1B). The S/B ratio decreased drastically for higher concentrations due to saturation of the activated surface with the capture antibody molecules generating sterical hindrance when interacting with the target protein. The S/B ratio value decreased with incubation times longer than 60 min, which is related to the poor antibody-target protein interaction [21]. Fig. S1C shows the results obtained using different activation reagents for the Ab anchorage. A higher S/B ratio was found for the covalent coupling of the IL6-Ab with an EDC/NHS mixture, as both reagents are necessary to increase the efficiency and stability of the formed ester-antibody complex. Fig. S2A shows a better S/B ratio when using ETA as a blocking reagent. All other blocking reagents tested gave worse S/B ratios due to possible secondary interactions between the blocking agent and IL6 protein. Fig. S2B shows a better S/B ratio when non-specific sites were blocked for only 15 min. Finally, the biorecognition event can be achieved in only 30 min (Fig. S2C). Table S2 (in the Supporting Information) summarizes the obtained results for the optimized variables.

Analogously, the experimental variables used to prepare a sandwich-like immunosensor were optimized. These conditions included the concentration of B-IL6-Ab and the incubation time with activated SPCE/pABA/neutravidin for its immobilization, the concentration of biotin used as the blocking reagent and the corresponding incubation time, as well as the incubation time in the IL6 protein standard solution for the formation of the sandwich-like immunosensor. Figs. S3 to S6 (in the Supporting Information) show the chronoamperometric responses and the corresponding S/B ratios. Fig. S3A shows a larger S/B ratio for 3 μg/mL of B-IL6-Ab solutions incubated for 60 min (Fig. S3B). The decrease in the antigen–antibody recognition response for higher concentrations is associated with steric hindrance caused by the capture antibody molecules that limit the interaction with the target protein. Figs. S4A-B show the results obtained to evaluate the biotin concentration and the corresponding incubation time. A concentration of 2 mg/mL and an incubation time of 30 min were selected to block non-specific sites during antigen–antibody recognition. In addition, the capture of the IL6 protein by B-IL6-Ab was carried out in only 45 min (Fig. S4C). Since amplification of the electrochemical signal was proposed to improve the analytical performance of the immunosensor, the concentrations of IL6-Ab and HRP in the employed nanobiohybrid were checked, as well as the concentration of the nanohybrid and the corresponding incubation time. Fig. S5A-B shows higher S/B ratios for 5 µg/mL and 1 mg/mL of IL6-Ab and HRP enzyme, respectively. Larger values affected the recognition of the IL6 protein due to the steric hindrance of the label. The optimization of the amount nanohybrid and incubation time yielded values of 0.01 µg and only 15 min as sufficient to achieve a better analytical performance of the immunosensor (Fig. S6A-B). Finally, the role of the hierarchical Au@Pt metal particles and the storage stability of the label (Fig. S7) were tested. A higher S/B ratio was achieved by incorporating Au@Pt nanoparticles on the surface of the MWCNTs (Fig. S7A), which can be attributed to the conductive and catalytic properties of both Au and Pt components, thus promoting faster electron transfer and improving the immunosensor response. Moreover, it was observed that after 10 days of nanobiohybrid storage with stirring at 4 °C, the response of the resulting immunosensor decreased (Fig. S7B). It may be due to the leaching of the nanobiohybrid components caused by the storage condition medium. Table S3 (in the Supporting Information) summarizes the optimized variables for the sandwich-like immunosensor.

### Analytical characteristics

**Fig. 3:**
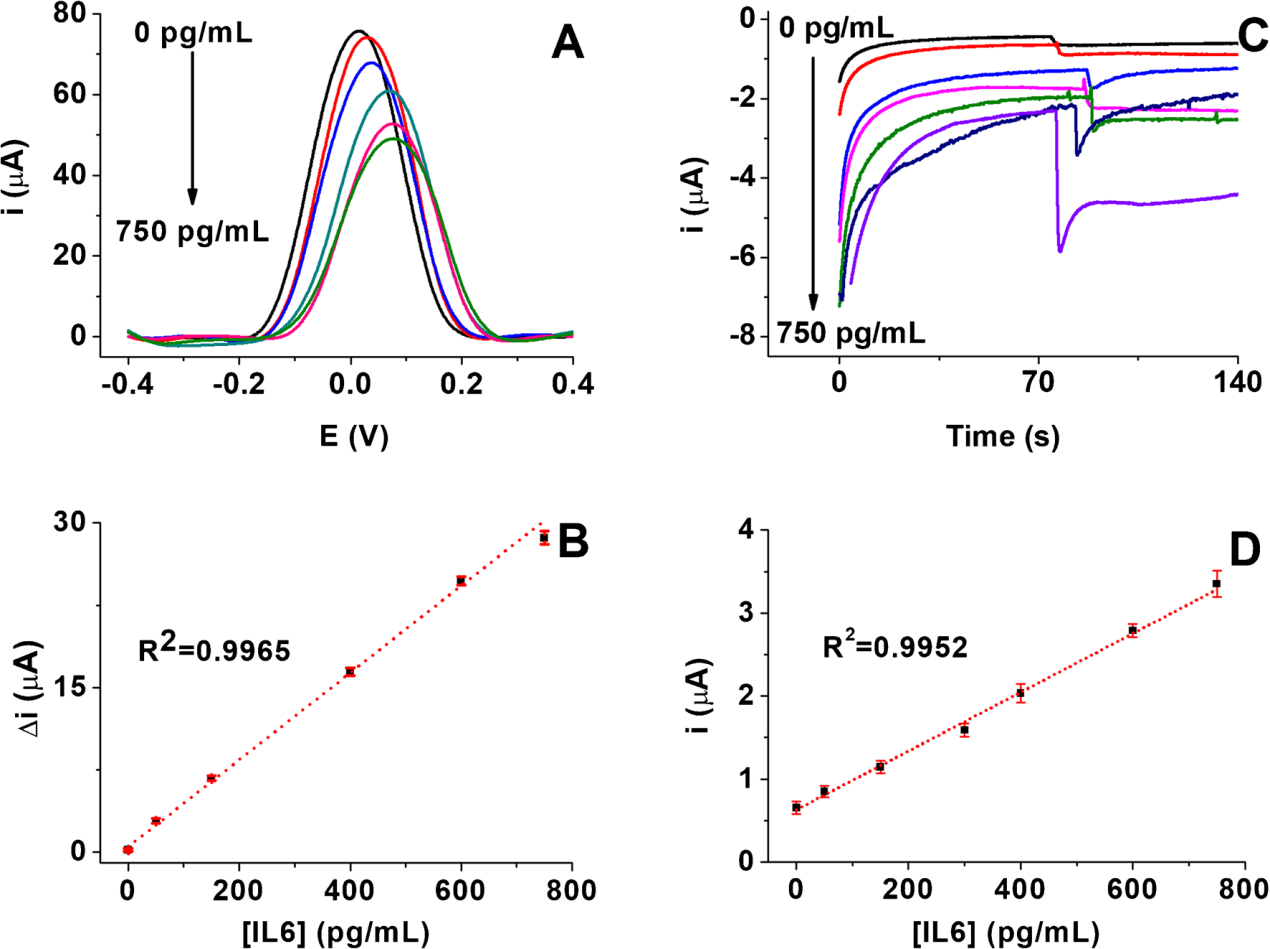
Electrochemical responses and corresponding calibration plots constructed for IL6 standard solutions (0 − 750 pg/mL in PBS 1X buffer pH 7.4) with the label-free inmunosensor by DPV using 5 mM [Fe(CN)_6_].^4−/3−^ as an electrochemical probe (**A**, **B**), and with the sandwich-type immunosensor by chronoamperometry with a detection potential of − 200 mV *vs.* the Ag *pseudo*-reference electrode and using the HQ/H_2_O_2_ system (**C**, **D**). Error bars were estimated as triple the standard deviation (*n* = 3)

**Fig. 4:**
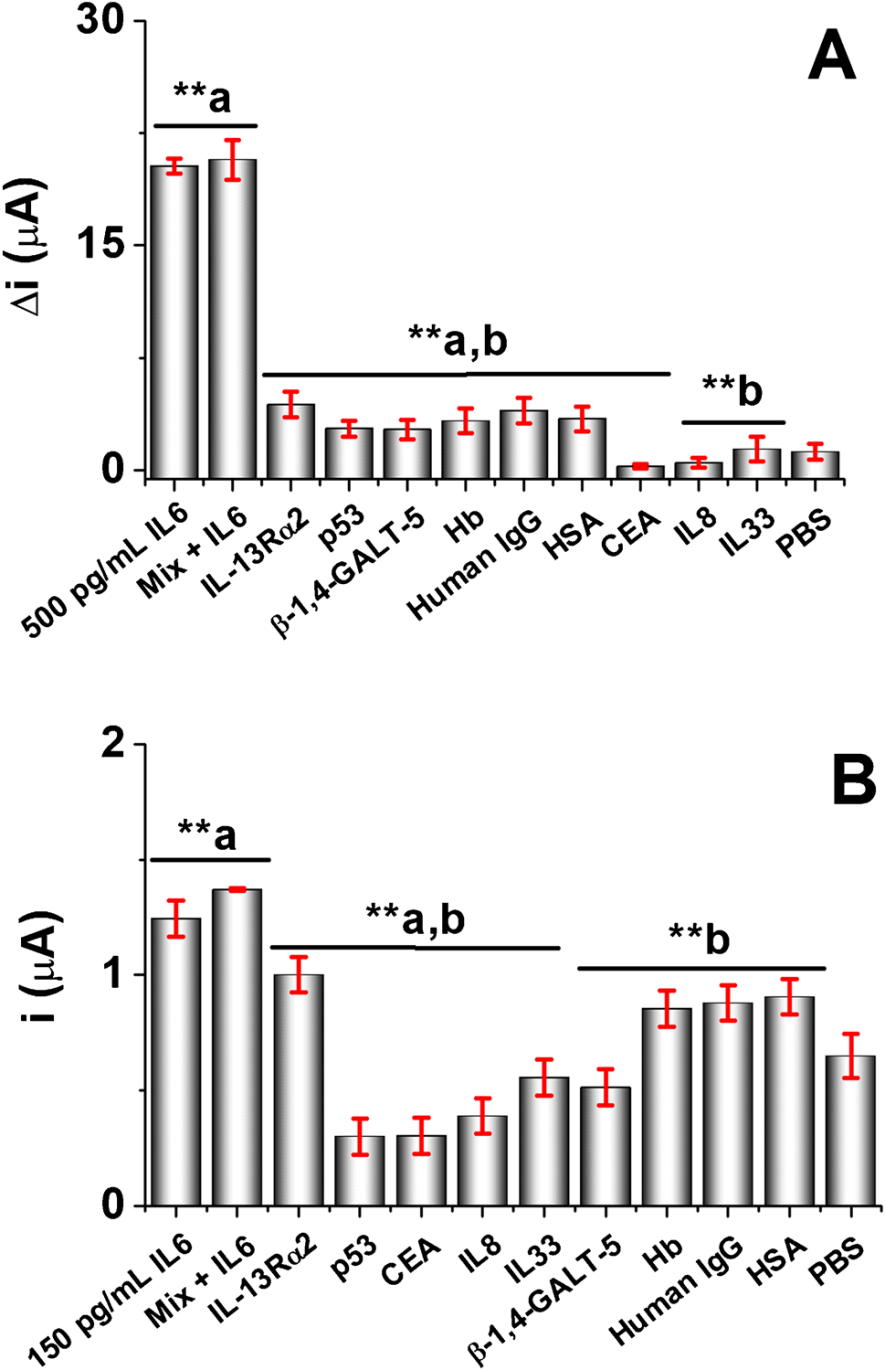
**A** DPV response for the label-free immunosensor in the presence of 500 pg/mL IL6 standard solution, and **B** amperometric response for the sandwich-like immunosensor in 150 pg/mL IL6 standard solution, both prepared in the absence and the presence of 50 ng/mL IL-13Rα2, 5.0 µg/mL p53, 1.0 µg/mL β-1,4-GALT-5, 0.5 mg/mL Hb, 0.1 mg/mL human IgG, 5.0 mg/mL HSA, 50 ng/mL CEA, 50 ng/mL IL8, and 35 ng/mL IL33. (**a) Significantly different concerning the PBS blank (*p* < 0.05). (**b) Significantly different concerning the IL6 protein (*p* < 0.05). (**a,b) Significantly different to PBS blank and 500 pg/mL IL6 protein (*p* < 0.05) for **A** or 150 pg/mL IL6 sample (*p* < 0.05) for **B**. Error bars were estimated as triple the standard deviation (*n* = 3)

Fig. 4 A shows the DPV responses for different IL6 glycoprotein concentrations ranging between 50 and 750 pg/mL. Figure 3 B displays the corresponding dependence of Δ*i* with IL6 concentrations as described by the equation Δ*i* (nA) = 40.00 [IL6] + 480.00 with a correlation coefficient *R*^2^ = 0.9965. The LOD and limit of quantification (LOQ) values of the immunosensors were calculated to be 14.4 and 47.9 pg/mL, respectively, as specified in the section “Evaluation of the analytical parameters” in the Supporting Information. The incorporation of a hybrid nanomaterial as a label provoked an enhancement in the analytical performance of the resulting electrochemical immunosensor (Fig. 3C). So, LOD and LOQ values of 6.0 pg/mL and LOQ of 20.0 pg/mL, respectively, were now achieved. The constructed calibration plot over the same linear range was fitted (*i *(nA) = 3.54 [IL6] + 629.00, *R*^2^ = 0.9952, Fig. 3D). The label in the sandwich-like immunosensor not only increased the sensitivity but decreased 2.4-fold the LOD with respect to the label-free format. This fact reveals the advantage of using labels for signal amplification, even considering the higher sensitivity of DPV versus chronoamperometry. As it is well known, chronoamperometry depends on H_2_O_2_/HQ diffusion towards the electrode surface and subsequent transfer of electrons related to the recognition of the IL6 protein by the nanohybrid system. In this work, we used a stirring system in the electrochemical cell, which may disturb the label’s integrity, causing sensitivity loss due to the leaching of the nanohybrid components. In addition, the label-free immunosensor is highly sensitive to changes at the interface, *i.e.*, slight changes in the concentration of salts in the buffers, electrolyte concentration, or pH can disturb the electrochemical response and generate a false signal. Regarding the sandwich-like immunoplatform, the presence of Pt in the nanoparticles generated high background signals since it also catalyzes the H_2_O_2_ reduction reaction.

The reproducibility of measurements provided by both labeled and label-free electrochemical immunosensors for 600 pg/mL IL6 standards was tested. Relative standard deviation (RSD) values of 2.7% and 1.6%, respectively, were obtained from the measurements carried out with three different immunoplatforms prepared following the specific protocols, allowing to conclude the whole operational reproducibility, including both the bioplatform preparation protocol and electrochemical transduction.

Significantly, levels of IL6 in serum of healthy individuals have been documented as low as 0.23–1.5 pg/mL compared to the higher reported range (1.9–130 pg/mL) in cancer patients [8, 38, 39]. Therefore, both immunosensor formats achieved a LOD and linear dynamic range suitable to measure overexpressed target glycoprotein levels [8, 40, 41]. Remarkably, the sensitivity of the immunosensors was improved by incorporating conductive nanomaterials on the electrode surface or as the label for amplification of the resultant signal. The high surface area of nanomaterials enables the immobilization of many bioreceptors, and their conductive properties improve the electrochemical performance, resulting in better immunosensor sensitivity and lowering the LOD and LOQ values.

Although numerous electrochemical immunosensors for the determination of IL6 are documented in the literature (see Table 1), none of them detected IL6 exploiting hierarchical Au@Pt nanoparticle/amino benzoic acid polymer–based hybrid materials and compared its use for label and label-free detection, which is the main novelty of this work. In addition, the nanohybrid was straightforward to manufacture compared to other processes, such as the development of microfluidic magnetoimmunosensors involving AuNP-graphene hybrids or the synthesis of interconnected hybrids using thionine as the anchor in AuNP-thionine-CMWCNTs [42].

**Table 1:** Comparison of the performance exhibited by nanomaterials or polymer-based electrochemical immunosensors/immunoassays for the determination of IL6

Immunosensor components	Rationale	Electrochemical technique	Analytical characteristics		Time it takes for the preparation of nanomaterials, the immunosensor/immunoassay and the determination	Sample	Ref
			Linear range (pg/mL)	LOD (pg/mL)			
Flexible BSA/cAb/Au/CF	Label-free direct immunosensor	DPV	0.001 − 1.5 × 10^6^	5.6 × 10^−5^	CFs: ~ 16 h Immunosensor: ~ 5 h Determination: ~ 15 min	Blood samples from PCa patients	[7]
BSA/cAb/ERGO-AuPdNPs/HCPE	Direct competitive immunosensor using IL-6-PS@PDA-AgNPs as nanolabels	LSV	0.1 − 100,000	0.059	Nanolabel: ~ 59 h Immunosensor: ~ 13 h Determination: ~ 40 min	Real serum samples	[43]
ITO/AB/EpxS-PPyr/IL6R/BSA	Label-free direct immunosensor	EIS	0.01 − 50	0.0032	Immunosensor: ~ 1 h Determination: ~ 45 min	Real serum samples	[44]
AuNPs/rGO/Au	Sandwich immunoassay onto MBs	Chronoamperometry	0.97 − 250	0.42	Determination: ~ 12 h	Spiked human serum samples	[45]
Au wire-ph-GO-cAb	Label-free sandwich immunosensor using dAb-GO-NB as nanolabels	SWV	1 − 300	1	Nanolabel: ~ 4 h Immunosensor: ~ 3 h Determination: ~ 1 h	Secretomes of RAW cells and secretion in vivo in the mouse brain	[46]
cAb/SPA/AuNPs-Thi-CMWCNTs/GCE	Label-free direct immunosensor	SWV	10–800,000	2.87	AuNPs-Thi-CMWCNTs: ~ 22 h Immunosensor: ~ 2 h Determination: ~ 25 min	Serum and different tissue lapping liquids (lung, heart, liver) from rats with myocardial infarction	[42]
ITO/PPCE/IL6R/BSA	Label-free direct immunosensor	EIS	0.02–16	0.006	Immunosensor: ~ 2 h Determination: ~ 30 min	Real serum samples	[47]
cAb/Au@Pt/pABA/SPCE	Label-free direct immunosensor	DPV	50 − 750	14.4	Immunosensor: ~ 2 h Determination: ~ 45 min	Spiked human serum samples	This work
b-cAb/NA/pABA/SPCE	Sandwich immunosensor using MWCNTs/Au@Pt/HRP/dAb as nanolabels	Chronoamperometry		6.0	Nanolabel: ~ 31 h Immunosensor: ~ 2 h Determination: ~ 45 min		

*AB* acetylene black, *AgNP* silver nanoparticle, *AuPdNPs* gold–palladium bimetallic nanoparticles, *Au-ph-GO* gold-4-aminophenyl phosphorylcholine-graphene oxide, *cAb* capture antibody, *BSA* bovine serum albumin, *CF* carbon fiber, *CMWCNTs* carboxylated multi-walled carbon nanotubes, *dAb* detector antibody, *DPV* differential pulse voltammetry, *EIS* electrochemical impedance spectroscopy, *EpxS-PPyr* epoxy-substituted-poly(pyrrole) polymer, *ERGO* electrochemically reduced graphene oxide, *GCE* glassy carbon electrode, *HCPE* heated carbon paste electrode, *IL-6R* interleukin-6 receptor, *ITO* indium tin oxide, *LSV* linear sweep voltammetry, *MBs* magnetic beads, *NA* neutravidin, *NB* Nile Blue, *pABA* p-aminobenzoic acid, *PCa* prostate cancer, *PDA* polydopamine, *PPCE* polypyrrole polymer containing epoxy active side groups, *PPyNPs* polypyrrole nanoparticles, *rGO* reduced graphene oxide, *SPA* Staphylococcal protein A, *SPCE* screen-printed carbon electrode, *SWV* square wave voltammetry, *Thi* thionine

### Selectivity and specificity studies

To check the immunosensor specificity, we evaluated the electrochemical response of the developed immunosensors to biomolecules that may cross-react with the anti-IL6 antibody or act as interferents in clinical samples. So, cytokines such as IL-13Rα2, IL33, and IL8; serum proteins such as hemoglobin (Hb), human IgG, and human serum albumin (HSA); non-target glycoproteins like β-1,4-GALT-5 and CEA; and tumor proteins such as p53 protein were tested at the concentrations indicated in Fig. 3 caption. Figure 3 A shows that the DPV response for IL6 was statistically significantly different (*p* < 0.05) with respect to all other molecules evaluated, indicating a high specificity with a significance level of 95% when analyzed by a paired *t* test and a one-way ANOVA. Furthermore, the Δ*i* value for Mix + IL6 was like for the IL6 signal, with no statistically significant differences, demonstrating the high selectivity of the label-free immunosensor able to discriminate the presence of IL6 in a sample coexisting with potential interferents. Moreover, some biomolecules did not show significant differences concerning PBS (*p* < 0.05), such as IL8 and IL33, confirming the high specificity of the immunosensor for IL6 (see Fig. 3A(**b)).

On the other hand, Fig. 3B shows a higher current response for the IL6 glycoprotein (*i* = 1.2 ± 0.1 µA) compared with other proteins such as p53 (*i* = 0.3 ± 0.1 µA), CEA (*i* = 0.3 ± 0.1 µA), IL8 (*i* = 0.4 ± 0.1 µA), β-1,4-GALT-5 (*i* = 0.5 ± 0.1 µA), and IL33 (*i* = 0.6 ± 0.1 µA), with statistically significant differences (**b: *p* < 0.05). However, no statistically significant differences were found when interrogating the PBS blank concerning Hb (*i* = 0.8 ± 0.1 µA), human IgG (*i* = 0.9 ± 0.8 µA), and HSA (*i* = 0.9 ± 0.1 µA). Furthermore, interrogation of the IL6 glycoprotein in the presence of all the other biomolecules derived in a slight response increase (*i* = 1.4 ± 0.1 µA). This result was attributable either to the slight non-specific adsorption of high-weight biomolecules such as IL-13Rα2 receptor, Hb, human IgG, and HSA or to some cross-reactivity by the B-IL6-Ab employed in the sandwich-type assembly (see Fig. 3B(**b)).

Therefore, although the sandwich-like format exhibited a lower LOD than the label-free counterpart, the label-free immunosensor is a simple-to-assemble platform with better selectivity than the sandwich-like immunosensor.

Furthermore, some studies report that nanobiohybrids have limited long-term stability, for instance, when immersed in PBS or chemical media for long periods [48]. Therefore, to evaluate its long-term stability, the nanobiohybrid was immersed in PBS pH 7.4 to preserve the biological activity of the antibodies and the enzyme, and the stability versus the freshly prepared nanobiohybrid was evaluated (see Fig. S7 in the Supporting Information). It was evident that there was a significant decrease in current intensity after the 10th day of storage. The nanohybrid-based label stability may be affected by the formation of oxide layers on the nanomaterial and the loss of biomolecules activity, thereby impacting its performance when assembled on the surface of the sandwich-like immunosensor.

### Quantification of IL6 in spiked human serum

The feasibility of using the developed immunosensors to determine IL6 was tested by analyzing spiked sera. The determination in this type of sample implied a 100-fold dilution to avoid any matrix effect while keeping the electrochemical responses within the linear ranges of the calibration curves obtained with IL6 standard concentrations (Fig. 4 B and D). Therefore, IL6 in the serum was determined by a simple interpolation of the electrochemical responses recorded for the samples into the corresponding calibration plot. Recoveries higher than 95% were achieved. The results reveal a high similarity in terms of recovery with both electrochemical immunosensors and suggest that the choice between the two immunosensors depends on application-specific considerations rather than inherent differences in recovery effectiveness. The obtained results are summarized in Table 2.

**Table 2:** Recovery assay in human serum spiked with IL6 standard solutions

Label-free immunosensor	Added [IL6] (pg/mL)	Recovered [IL6] (pg/mL)	Recovery (%)	RSD (%) (*n* = 3)
	–	N.D		
	150	149.6	99.7	4.1
	300	283.9	94.6	3.6
	600	586.1	97.7	3.2
	750	756.2	100.8	4.2
Sandwich-like immunosensor	Added [IL6] (pg/mL)	Recovered [IL6] (pg/mL)	Recovery (%)	RSD (%) (*n* = 3)
	-	N.D		
	150	144.3	96.2	3.4
	300	290.3	96.8	3.2
	600	597.2	99.5	2.6
	750	770.3	102.7	4.6

*N.D.* non-detectable

On the other hand, the outstanding reproducibility observed with the implemented electrochemical immunoplatforms underlines their robustness and measurement reliability. This crucial attribute strengthens the validity and generalizability of the presented results, opening new opportunities for applying this technology in a wide range of biomarkers.

The developed immunosensors are promising tools in the diagnosis/prognosis of cytokines involved with inflammatory processes in various diseases in a decentralized environment, with minimal patient sample handling and rapid response. Further work is directed toward a complete validation procedure for the immunosensors, analyzing a statistical number of positive and negative samples to assess the device’s true potential as an analytical tool for detecting IL6 in patient samples.

## Conclusions

A comparative evaluation of labeled and label-free electrochemical immunosensors based on a hybrid carbonaceous polymeric material with Au@Pt nanoparticles for the selective and specific detection of IL6 is reported. After an exhaustive optimization process, the analytical performance of the resulting electrochemical immunosensors was evaluated by DPV and chronoamperometry, respectively. It was found that using the labels presents some limitations in the development process and the analytical performance. For example, the label entails a longer preparation time and presents low storage stability (10 days) and lower specificity than the label-free immunosensor, increasing costs and the response time of the amperometric signal. However, it presents a LOD and linear range comparable with other reports and within the ranges of clinical interest for detecting overexpressed IL6 typical of diseases such as cancer. It should also be noted that chronoamperometric transduction using the sandwich-type immunosensor is more straightforward to implement in POC devices. On the other hand, the label-free immunosensor exhibits better specificity, and measurements can be taken 45 min after the immobilization of the capture antibody and blocking steps (≈ 2 h in both formats). However, its LOD is higher than that of the labeled immunosensor, impacting its practical applicability in the detection of overexpressed IL6 in diseases such as cancer. Overall, the results showed that introducing metallic hybrid nanomaterials as modifiers of transducer platforms or as labels for signal amplification are plausible alternatives for designing immunosensors with outstanding analytical performance. The resulting immunosensors could also be versatile tools to detect IL6 and other biomarkers related to the diagnosis/prognosis of immune-mediated diseases and cancer. Therefore, it can be concluded that the results presented in this work can be considered as a good starting point to continue exploiting the potential of this new hybrid nanomaterial with great versatility of modification and use in the development of immunoplatforms based on other formats (for example, indirect competitive configuration by immobilizing the target antigen on Au@Pt/pABA/SPCE or direct competitive format by immobilizing the target antigen and HRP on MWCNTs/Au@Pt). This new material may also be employed for the development of other affinity bioplatforms besides immunosensors, such as nucleic acid, peptide or aptameric bioplatforms, and for the development of multiplexed and/or multiomic biotools taking advantage of the versatility of the developed nanomaterial and substrates, and electrochemical instrumentation.

## Supplementary Information

Below is the link to the electronic supplementary material.

Supplementary file1 (DOCX 1.65 MB)

## Data Availability

No datasets were generated or analysed during the current study.
