# Supplementary material for: Hierarchical Au@Pt nanoparticle/amino benzoic acid polymer-based hybrid material for labeled and label-free detection of interleukin-6: a comparative assessment
Source: Mikrochim Acta. 2024 Oct 21;191(11):683. doi: 10.1007/s00604-024-06745-y (PMC11493819; doi:10.1007/s00604-024-06745-y)
Supplement: Supplementary file 1 — Supplementary file1 (DOCX 1.65 MB) [file 604_2024_6745_MOESM1_ESM.docx]

**SUPPORTING INFORMATION**

**Hierarchical Au@Pt nanoparticle/amino benzoic acid polymer-based hybrid material for labeled and label-free detection of interleukin-6: a comparative assessment**

Dayana Soto^a#^, Verónica Serafín^b^, María Pedrero^b^, José M. Pingarrón^b^, Susana Campuzano^b^, Jahir Orozco^a*^

^a^Max Planck Tandem Group in Nanobioengieneering, University of Antioquia, Complejo Ruta N, Calle 67 Nº 52-20, Medellín, 050010, Colombia

^b^Departamento de Química Analítica, Facultad de CC. Químicas, Universidad Complutense de Madrid, Pza. de las Ciencias 2, 28040 Madrid, Spain

^#^Current filiation: CECOLTEC Group, Cecoltec Services S.A.S company, Universidad EAFIT, Edificio Ingenierías, Bloque 19, Medellín, 050022, Colombia.

* To whom correspondence should be addressed (grupo.tandemnanobioe@udea.edu.co)

# Experimental

## Apparatus and electrodes

Voltammetric experiments were made with a potentiostat galvanostat VersaSTAT 3 with a single channel and an impedance mode using the VersaStudio 2.61.3 software or a CHI812B potentiostat (CH Instruments, Inc.) controlled by CHI812B software (amperometric measurements). Screen-printed carbon electrodes (SPCEs, DRP-110, Metrohm DropSens) consisting of a 4-mm diameter carbon working electrode, a carbon counter electrode, and an Ag *pseudo*-reference electrode were used as electrochemical transducers. All measurements were performed at room temperature.

The morphology and composition of the hybrid nanomaterials were analyzed by scanning electron microscopy (SEM), an energy-dispersive X-ray spectroscopy (EDX) system, and Apreo 2 SEM microscope operating at an accelerating voltage of 30 keV. Images were analyzed using Image J software.

## Reagents and solutions

Recombinant human interleukin-6 (IL6) was obtained from the Human Total IL6 DuoSet^®^IC ELISA kit (R&D Systems, Inc. Catalog Number DY206-05) containing mouse anti-human IL6 antibody (IL6-Ab), biotinylated goat anti-human IL6 antibody (B-IL6-Ab) and standard recombinant human IL6. The main reagents, including citric acid, potassium ferricyanide (K_3_[Fe(CN)_6_]), and potassium hexacyanoferrate trihydrate (K_4_[Fe(CN)_6_]×3H_2_O), were purchased from Merck Millipore. Dipotassium hydrogen phosphate (K_2_HPO_4_), disodium hydrogen phosphate (Na_2_HPO_4_), and potassium nitrate (KNO_3_) were acquired from PanReac AppliChem. Potassium dihydrogen phosphate (KH_2_PO_4_), potassium chloride (KCl), and sodium chloride (NaCl) were obtained from J.T.Baker^®^. Sulphuric acid (H_2_SO_4_) was purchased from Honeywell FlukaTM. Pierce™ Protein-Free PBS (Ref. 37572, protein-free blocking buffer, PFBB solution) was acquired from Thermo Scientific. Besides, bovine serum albumin (BSA, Cat. No.: 90604-29-8) was from Gerbu Biotechnik. Horseradish peroxidase (HRP) was obtained from Calbiochem. The IL6 antibody solution was prepared in commercial blocker™ casein in PBS blocking buffer (BB) (Cat. No: 37528 ThermoFisher Scientific). Human hemoglobin (Hb, Cat. No.: H7379), IgG standards from human serum (Human IgG, ≥ 95 %, Cat. No.: I2511), albumin from human serum (HSA, ≥ 96 %, Cat. No: A1653), hydroquinone (HQ), hydrogen peroxide (H_2_O_2_, 30 % v/v), 4-aminobenzoic acid (ABA), multi-walled carbon nanotubes (MWCNT ∼95% purity assay; outer diameter size, 10–15 nm; inner diameter size, 2–6 nm; length, 0.1–10 mm), ethanolamine (ETA), N-(3-dimethylaminopropyl)-N′-ethylcarbodiimide hydrochloride (EDC), N-hydroxysuccinimide (NHS), pluronic® F127 (F127), sodium citrate tribasic dihydrate, tetrachloroauric acid trihydrate (HAuCl_4_.3H_2_O) and hexachloroplatinic acid hexahydrate (H_2_Cl_6_Pt×H_2_O) were purchased from Sigma-Aldrich. Human interleukin-33 (IL33, Cat. No: RAB0297), Interleukin-13 receptor subunit alpha-2 (IL-13Rα2, Cat. No: DY614), carcinoembryonic antigen (CEA, Cat. No: C4835), recombinant cellular tumor antigen p53 protein (p53, Cat. No: ab199593), human interleukin-8 (IL8, Cat. No: ab48481) and β-1,4-galactosyltransferase-V glycoprotein (β-1,4-GALT-5, Cat. No: ab16047) were purchased from Abcam. They were tested as potential interfering species to evaluate the specificity of the developed immunosensors.

Phosphate buffer saline (PBS) consisting of 0.01 mol/L phosphate buffer solution containing 0.137 mol/L NaCl and 0.0027 mol/L KCl, pH 7.5; and 0.05 mol/L phosphate buffer (PB), pH 6.0; 25 mmol/L MES buffer, pH 5.0, were used. All chemical reagents used were of analytical grade, and all aqueous solutions were prepared with ultrapure water (18.2 MΩ cm) from a Milli-Q™ Element A10 System.

## Nanobiohybrid synthesis and assembly of the immunosensors

Regarding the label-free immunosensor, SPCEs were modified by cyclic voltammetry (CV) electropolymerization of a 4-aminobenzoic acid (ABA) solution until generating a thin pABA layer, followed by electroplating the hierarchical nanoparticles (Au@Pt) on the modified electrodes through CV. The modified surface was activated by adding 6.0 µL of a 100 mM EDC/NHS mixture in 25 mM MES (pH 6.5) and incubating for 30 min. Then, Ab solution was incubated for 1 h at 25 ºC in a wet chamber and washed to remove the not anchored receptor, followed by incubation with 1 M ETA solution for 15 min at 25 ºC as a blocking agent of non-specific sites. The immunosensor was then incubated in an IL6 glycoprotein solution at 25 ºC for 45 min in the wet chamber for the antibody-protein interaction.

The sandwich-like immunosensor uses two antibodies to detect the target analyte. For this purpose, the MWCNTs-based label was first synthesized as follows. 1.5 mg of MWCTNs were dispersed in 1 % sodium citrate by sonication for 5 min. Subsequently, the mixture was heated at 30 °C with 30 mM HAuCl_4_×3H_2_O, 3 mM H_2_Cl_6_Pt.×H_2_O, and 14.2 mM F127 for 20 min in a water bath. The obtained material was washed and dispersed in water. For immobilizing HRP and IL6-Ab on the MWCNTs, the MWCNTs surface was previously activated with a mixture of 400 mM EDC and 100 mM NHS in 100 mM PBS pH 7.4 for 6 h at 4 °C. The MWCNTs dispersion was centrifuged and washed with 100 mM PBS pH 7.4 and then led to interact with 1 mg/mL HRP and 240 µg/mL Ab for 24 h at 4 °C. Finally, the nanobiohybrid was washed with PBS pH 7.4 to remove unanchored HRP and IL6-Ab molecules and dispersed in PBS pH 7.4.

The SPCE was activated by adding 6.0 µL of a mixture of 100 mM EDC/NHS in 25 mM MES (pH 6.5) and incubation for 30 min; then, 600 µg/mL neutravidin in 25 mM MES (pH 6.5) was anchored to the modified electrode surface. Next, the B-IL6-Ab solution was incubated in the wet chamber for 1 h at 25 ºC and washed to remove the not anchored receptor, followed by adding 2 mg/mL biotin solution for 30 min at 25 ºC to block non-specific sites. The resulting immunosensor was incubated in an IL6 protein solution at 25 ºC for 45 min in the wet chamber to allow the antibody-protein interaction. Finally, 0.01 µg of nanobiohybrid were added for signal amplification and incubated for 15 min.

## Optimization of experimental parameters

The experimental conditions used to assemble the immunosensors were selected according to a better signal/blank (S/B) ratio. With the label-free immunosensor, the S/B ratio was calculated from the DPV immunosensor responses in buffered solutions without and with 600 pg/mL IL6 protein. Ab concentrations of 0, 25, 50, 100, and 200 μg/mL with 60 min incubation and IL6 incubation times over the 0 to 60 min range were tested.

Similarly, for the sandwich-like immunosensor, chronoamperometric responses in the absence and the presence of 600 pg/mL IL6 were compared. B-IL6-Ab concentrations of 0, 1.5, 3.0, 4.5, and 6.0 μg/mL and IL6 incubation times from 15 to 60 min were checked.

## Electrochemical measurements

The SPCE was modified as described in the "Nanohybrid synthesis and assembly of the immunosensors" section, and the immunoplatform was characterized with electrochemical techniques. CV and electrochemical impedance spectroscopy (EIS) responses using the 5 mM [Fe(CN)_6_]^4-/3-^ redox pair in PBS 1X pH 7.4 as supporting electrolyte were recorded for each step of the immunosensor assembly. The potential window for CV was between + 0.4 V and ‒ 0.2 V at a scan rate of 0.05 V/s for five consecutive cycles. This measurement was made initially with the bare electrode and subsequently with each step of the functionalization process. The formal potential of the redox pair (+ 0.18 V) was applied to carry out the EIS measurement. The amplitude of the sinusoidal wave was 10 mV, and frequencies ranged from 100 kHz to 0.01 Hz. Using the EIS analyzer software to characterize the interfacial electrical properties, the EIS data were fitted to the electrical equivalent circuit. EIS was used after each step of the immunosensor development, i.e., after the formation of the pABA polymeric layer, electrodeposition of Au@Pt nanoparticles, activation of the carboxyl groups, covalent coupling of the capture antibody, blocking of the remaining active esters, and incubation with the molecular target. Subsequent steps were fitted where *R_s_* is the solution resistance, *CPE* is the constant phase element, *R_ct_* is the charge transfer resistance, and *Z_w_* is Warburg's element.

DPV was used to evaluate the response of label-free immunosensors to different concentrations of IL6 standard solution. A 5 mM [Fe(CN)_6_]^4-/3-^ in PBS 1X pH 7.4 solution was dropped on a label-free immunosensor after the interaction of IL6 protein with the bioplatform. The response of the immunosensor was evaluated between + 0.5 V and − 0.3 V at an amplitude of 0.05 V for 0.05 s.

The sandwich-like integrated inmunoplatform was immersed in a measuring cell containing 10 mL of 50 mM PB buffer (pH 6.0) and 100 µL of freshly prepared 100 mM HQ in the same buffer kept under constant gentle stirring and using a detection potential of – 200 mV vs. the Ag *pseudo*-reference electrode. Once the background current was stabilized (approximately 60 s), 50 µL of the 100 mM H_2_O_2_ substrate solution freshly prepared in 50 mM PB buffer (pH 6.0) was added to the measuring cell, and the cathodic current variation occurring by the HQ reduction in the presence of HRP and H_2_O_2_ was recorded until reaching the steady state. Amperometric responses were calculated as the difference between the steady state and the background currents, i.e., the responses correspond to the cathodic current variations obtained in the presence and the absence of IL6 (Δi).

## Evaluation of the analytical parameters

Once the optimal conditions were determined, the analytical performance of the electrochemical immunosensors was evaluated, measuring different concentrations of the IL6 protein. Different solutions with known IL6 protein concentrations were measured with label-free and sandwich-like immunosensors by DPV and chronoamperometry, respectively. The calibration curve for IL6 protein using the label-free immunosensor was constructed by plotting Δi versus IL6 protein concentration (0 to 750 pg/L in buffer PBS 1X pH 7.4). Changes in the current intensity were calculated according to Δi = i(ETA) ‒ i(IL6).

For the sandwich-like immunosensor, chronoamperometry measurements for IL6 solutions of different concentrations were used to quantify changes in IL6 protein concentration. The resulting IL6 protein solutions were analyzed with three independent immunosensors in both cases. The limit of detection (LOD) and limit of quantification (LOQ) of the immunosensors were calculated by using the 3-sigma and 10-sigma criterion, respectively, estimated according to LOD = 3 σ_B_/m and LOQ = 10 σ_B_/m, where σ_B_ was the standard deviation of 10 measurements obtained in the absence of IL6 protein, and m the slope of the respective calibration plot.

## Selectivity studies

To assess the selectivity of the developed electrochemical immunosensors, we evaluated the cross-reactivity of the IL6 antibody separately to 0.5 mg/mL Hb, 0.1 mg/mL human IgG, 5.0 mg/mL HSA, 35 ng/mL IL33 protein, 50 ng/mL IL-13Rα2, 50 ng/mL CEA, 5.0 µg/mL p53, 50 ng/mL IL8 protein, and 1.0 µg/mL β-1,4-GALT-5 glycoprotein. Such biomolecules are overexpressed in inflammatory processes and may be cross-reactive with the IL6 antibody. In addition, IgG and hemoglobin are abundant biomolecules in human serum and may also interfere in determining the IL6 protein. In addition, studies were made by mixing 500 pg/mL (label-free immunosensor) or 150 pg/mL (sandwich-like immunosensor) of IL6 protein with all the potential interferents mentioned above. A paired t-test and a 1-way ANOVA with a 95 % level of statistical significance were performed to evaluate differences among the samples.

## Quantification of IL6 in serum samples

As a proof of concept, both electrochemical immunosensors were used to determine IL6 in commercial human serum. This was spiked with known concentrations of IL6 (150, 300, 600, and 750 pg/mL) in PBS pH 7.4, containing 0.1 % Tween-20 (v/v) and 3 % BSA (w/v). The resultant solutions were vortexed for 15 s and incubated at 37 ºC for 15 min to dissolve the possible lipid aggregates in the matrix. Next, the insoluble residual components of the sample were removed by centrifugation at 13,000 rpm and 4 °C for 5 min. Thereafter, 6 μL of supernatant were dropped onto the SPCE and incubated in the wet chamber for 45 min to bind the IL6 protein. Next, three washing steps with ultrapure water were performed to remove the components that did not bind to the antibody. Finally, the analyses were completed using the abovementioned protocol to determine the IL6 protein by DPV and chronoamperometry, depending on the immunosensor format.

**Results and discussion**

**Table S1.** EIS characterization of the stepwise preparation of the developed immunosensors for IL6. Charge-transfer resistance (R_ct_), electrolyte solution resistance (R_s_), Warburg impedance (Z_W_), constant phase element (CPE) with pre-exponential factor (P) and exponent (n) and Chi-squared function (χ2).

| **Immunosensor** | **Electrode** | **R_ct_ (Ω)** | **R_s_ (Ω)** | **Z_W_ (Ω s^−0.5^)** | **CPE** | | **χ^2^** |
| --- | --- | --- | --- | --- | --- | --- | --- |
|  |  |  |  |  | **P (Ω^−1^ s^n^)** | **n** |  |
| **Label-free** | **SPCE bare** | 1707.9 ± 0.1 | 402.2 ± 0.1 | 1231.1 ± 0.1 | 2.83×10^−6^ | 0.89 | 1.03×10^−3^ |
|  | **pABA** | 8988.5 ± 0.2 | 409.0 ± 0.3 | 880.7 ± 0.1 | 1.01×10^−6^ | 0.76 | 3.47×10^−3^ |
|  | **Au@Pt** | 104.5 ± 0.1 | 466.8 ± 0.4 | 1932.1 ± 0.3 | 2.02×10^−6^ | 0.89 | 1.52×10^−3^ |
|  | **NHS-EDC/IL6-Ab** | 1107.6 ± 0.1 | 473.2 ± 0.6 | 1825.1 ± 0.1 | 4.09×10^−4^ | 0.95 | 1.45×10^−3^ |
|  | **ETA** | 1392.9 ± 0.2 | 475.4 ± 0.5 | 1831.1 ± 0.4 | 2.50×10^−4^ | 0.96 | 1.24×10^−3^ |
|  | **IL6 protein** | 1992.9 ± 0.1 | 472.2 ± 0.1 | 1735.4 ± 0.3 | 2.00×10^−4^ | 0.94 | 3.43×10^−3^ |
| **Sandwich-like** | **SPCE bare** | 1707.9 ± 0.1 | 402.2 ± 0.1 | 1231.1 ± 0.1 | 2.83×10^−6^ | 0.89 | 1.03×10^−3^ |
|  | **pABA** | 8988.5 ± 0.2 | 409.0 ± 0.3 | 880.7 ± 0.1 | 1.01×10^−6^ | 0.76 | 3.47×10^−3^ |
|  | **NHS-EDC/neutravidin** | 1971.4 ± 0.1 | 517.3 ± 0.1 | 1298.1 ± 0.2 | 9.56×10^−5^ | 0.88 | 2.03×10^−3^ |
|  | **B-IL6-Ab** | 2012.7 ± 0.2 | 484.3 ± 0.3 | 1304.5 ± 0.3 | 7.68×10^−5^ | 0.98 | 1.08×10^−3^ |
|  | **Biotin** | 2945.2 ± 0.1 | 485.5 ± 0.1 | 1292.9 ± 0.1 | 1.44×10^−4^ | 0.91 | 1.75×10^−3^ |
|  | **IL6 protein** | 3082.9 ± 0.1 | 490.0 ± 0.1 | 1320.0 ± 0.3 | 9.76×10^−5^ | 0.92 | 1.46×10^−3^ |
|  | **Nanobiohybrid** | 2062.1 ± 0.3 | 488.1 ± 0.2 | 1331.8 ± 0.3 | 2.79×10^−4^ | 0.92 | 1.21×10^−3^ |

**Optimization of the experimental variables involved in the preparation and performance of the label-free immunosensor**

*a. IL6-Ab concentration and incubation time and effect of mixing the activation reagents*









**Fig. S1.** DPV responses for variations of A) IL6-Ab concentration, B) incubation time of IL6-Ab, and C) activation mixture for 0 (B) and 600 (S) pg/mL IL6 standard solutions. Error bars were estimated as triple the standard deviation (n=3).

*b. Effect of blocking reagent, incubation time with ETA and IL6.*









**Fig. S2.** DPV responses showing the effect of A and B) blocking reagent and incubation time with ETA; C) incubation time with IL6 for 0 (B) and 600 (S) pg/mL IL6 standard solutions. Error bars were estimated as triple the standard deviation (n=3).

**Table S2.** Experimental variables optimized in the preparation of the label-free immunosensor for IL6.

| **Variable** | **Evaluated range** | **Selected value** |
| --- | --- | --- |
| IL6-Ab concentration (µg/mL) | 0–200 | 100 |
| Incubation time with IL6-Ab (min) | 15–90 | 60 |
| Activation mixture | EDC/NHS or EDC | EDC/NHS |
| Blocking reagent | ETA-BSA-PFBB-MCH | ETA |
| Incubation time with the blocking reagent (min) | 0–90 | 15 |
| Incubation time with IL6 (min) | 0–60 | 45 |

**Optimization of the experimental variables involved in the preparation and performance of the sandwich-like immunosensor**

1. *B-IL6-Ab concentration and incubation time*







**Fig. S3.** Chronoamperometric responses obtained upon variation of A) B-IL6-Ab concentration and B) incubation time for 0 (B) and 600 (S) pg/mL IL6 standard solutions. Error bars were estimated as triple the standard deviation (n=3).

*b. Biotin concentration, biotin, and IL6 incubation times.*

*





*

**Fig. S4.** Chronoamperometric responses were measured when the A) biotin concentration, B) biotin incubation time, and C) IL6 incubation time were varied for 0 (B) and 600 (S) pg/mL IL6 standard solutions. Error bars were estimated as triple the standard deviation (n=3).

*c. Concentration of IL6-Ab and HRP in the nanobiohybrid*

*



*

**Fig. S5.** Chronoamperometric responses were measured to evaluate the change in the concentration of A) IL6-Ab and B) HRP for 0 (B) and 600 (S) pg/mL IL6 standard solutions. Error bars were estimated as triple the standard deviation (n=3).

*d. Nanobiohybrid concentration and incubation time*







**Fig. S6** Chronoamperometric responses obtained using different nanohybrid concentrations A) and incubation time B) for 0 (B) and 600 (S) pg/mL IL6 standard solutions. Error bars were estimated as triple the standard deviation (n=3).

*e. Stability of nanobiohybrid from the sandwich-type immunosensor*







**Fig. S7.** Chronoamperometric responses to evaluate the effect of A) label type and B) storage stability of the nanobiohybrid for 0 (B) and 600 (S) pg/mL IL6 standard solutions. Error bars were estimated as triple the standard deviation (n=3).

**Table S3.** Experimental variables optimized in the preparation of the sandwich-like immunosensor for IL6.

| **Variable** | **Range evaluated** | **Selected** |
| --- | --- | --- |
| B-IL6-Ab **c**oncentration (µg/mL) | 0.0–6.0 | 3.0 |
| B-IL6-Ab incubation time (min) | 15–90 | 60 |
| Biotin concentration (mg/mL) | 1.0–3.0 | 2 |
| Biotin incubation time (min) | 15–60 | 30 |
| IL6 incubation time (min) | 15–60 | 45 |
| IL6-Ab concentration (µg/mL) | 0.0–10.0 | 5.0 |
| HRP concentration (mg/mL) | 0.0–2.0 | 1.0 |
| Amount of nanobiohybrid (µg) | 0.00–0.02 | 0.01 |
| Nanobiohybrid incubation time (min) | 0–60 | 15 |
| Label components | MWCNT, Au, Pt | MWCNT/Au@Pt |
| Nanobiohybrid storage stability (days) | 0–20 | < 10 |
